# Anti‐ageing effects of red ginseng on female *Drosophila melanogaster*

**DOI:** 10.1111/jcmm.15029

**Published:** 2020-02-05

**Authors:** Wei Hou, Jin Pei, YingPing Wang, Jiao Zhang, HouSheng Zheng, Ranji Cui

**Affiliations:** ^1^ School of Pharmaceutical Sciences Jilin University Changchun China; ^2^ Institute of Special Animal and Plant Sciences Chinese Academy of Agricultural Sciences Changchun China; ^3^ Jilin Provincial Key Laboratory on Molecular and Chemical Genetic The Second Hospital of Jilin University Changchun China

**Keywords:** anti‐ageing, drosophila, life‐prolonging, proteomic, red ginseng

## Abstract

Red ginseng (RG) was recently reported to extend the lifespan of *Drosophila melanogaster*. However, the mechanism underlying this effect has not yet been elucidated. The present study aimed to elucidate the molecular mechanisms of the RG‐mediated prolongation of the lifespan of female *D melanogaster*. In this study, protein changes in 36‐day‐old female *D melanogaster* were identified using isobaric tag for relative and absolute quantitation (iTRAQ), and levels of differentially expressed proteins were verified by quantitative real‐time PCR and Western blotting. Our studies have shown that RG concentrations of 12.5, 15 and 17.5 mg/mL significantly prolonged the lifespan. Eleven proteins were up‐regulated and 46 were down‐regulated between the RG and control groups; and Pebp1 expression was significantly down‐regulated. In addition, AKT and p‐AKT were down‐regulated, and ERK, p‐ERK and Raf1 were up‐regulated by RG. Therefore, RG significantly prolonged the lifespan of female *D melanogaster* by reducing the expression of Pebp1, up‐regulating ERK and inhibiting the AKT pathway. RG may be a potential drug for anti‐ageing treatment.

## 1 INTRODUCTION

Ginseng (*Panax ginseng* Meyer, Araliaceous) is an important traditional medicinal herb that has been widely used for millennia. Red ginseng (RG) that is steamed and dried from fresh ginseng exerts a variety of physiological effects, which include prolonging the lifespan of *Drosophila melanogaster* and improving learning and memory.^1^, ^2^, ^3^ In our previous study, the expression of age‐related proteins in 21‐day‐old male *D melanogaster* (middle‐aged) with RG extract (RGE) treatment was studied using the 2D‐PAGE system.^1^ In this study, an integrated analysis of protein changes of 36‐day‐old female *D melanogaster* (old‐age) was performed using isobaric tag for relative and absolute quantitation (iTRAQ). The study of protein changes in old‐age *D melanogaster* was helpful to reveal the life‐prolonging and anti‐ageing effects of RG.

## 2 MATERIALS AND METHODS

### 2.1 Materials

RG (6 years) was obtained from Changchun (Jilin Province, China). The contents were as follows (all in mg/g): Re 0.25, Rg1 0.73, Ro 1.26, Rf 0.56, Rb1 4.37, Rc 2.55, Rb2 2.91, Rb3 0.48, Rd 1.59, Rg3(s) 0.14 and Rg3(r) 0.07.

### 2.2 Lifespan analysis of female *Drosophila melanogaster*

Wild‐type *D melanogaster* was obtained from Jilin Agricultural University (Changchun, China). Single populations (200 flies each) of control‐female *D melanogaster* were fed a basal food containing water. The RG group was fed the basal food supplemented with RG.

### 2.3 Protein preparation

*D melanogaster* (36 days old, 20 flies each) was anaesthetized and collected. Samples were ground into fine powder in liquid nitrogen and then dissolved in SDT buffer (4% sodium dodecyl sulphate, 0.1 mol/L; dithiothreitol, 100 mmol/L; and Tris‐HCl, pH 7.6). The peptides were desalted on MILI‐SPE Extraction disc cartridge (C18‐SD), lyophilized and added to 40 µL of dissolution buffer.

### 2.4 iTRAQ labelling

A peptide mixture (100 μg) of each sample was labelled using the iTRAQ reagent‐8 plex Multiplex Kit (AB SCIEX UK Limited). Control‐female‐1 (113 tag), control‐female‐2 (114 tag), control‐female‐3 (115 tag), RG‐female‐1 (116 tag), RG‐female‐2 (117 tag) and RG‐female‐3 (118 tag).

### 2.5 LC‐MS/MS proteomic analysis

Each sample was injected for nano LC‐MS/MS analysis coupled to an EASY nLC (Thermo Fisher Scientific). The sample was loaded into a Thermo Scientific Acclaim PepMap100 column (100 μm × 2 cm, nanoViper C18) using an automatic sampler and connected to an analytical column (Thermo Fisher Scientific EASY Column; 10 cm, ID75 μm, 3 μm, C18‐A2) in buffer A (0.1% formic acid) and buffer B (84% acetonitrile and 0.1% formic acid) at a flow rate of 300 nL/min. LC‐MS/MS analysis was performed using an Q Exactive mass spectrometer (Thermo Fisher Scientific).

### 2.6 Proteomic data analysis

Proteins were identified using the MASCOT engine (version 2.2; Matrix Science) embedded in Proteome Discoverer 1.4 (Thermo Fisher Scientific) against the database (UniProt *Drosophila melanogaster* 42524 20180327. fasta). Differentially expressed proteins were functionally annotated using the Blast2GO program (https://www.blast2go.com/). Pathway enrichment analysis of significant proteins was performed using the Kyoto Encyclopedia of Genes and Genomes (KEGG) database.^4^, ^5^ A q‐value was 0.01.

### 2.7 qRT‐PCR

Female *D melanogaster* (36 days old, 20 flies each) of the control and RG groups was anaesthetized and collected. Glyceraldehyde‐3‐phosphate dehydrogenase (GAPDH) was used as an internal reference gene. Total RNAs were extracted with UNIQ‐10 Trizol Total RNA Extraction Kit (SK1321) according to the manufacturer's instructions. The cDNA was synthesized using cDNA Synthesis kits (RevertAid Premium Reverse Transcriptase; EP0733, Thermo Fisher Scientific). RNA expression analysis was calculated using the 2^−ΔΔCT^ methods (Primer sequences of qRT‐PCT in Table 1).

**Table 1:** Primer sequences of qRT‐PCT

Gene	Primer sequences
*CG12895*	*F 5′ GTGTCAGCAACGACTGGGATA 3′*
	*R 5′ GCTCCTTTAGCAGACGCATTAT 3′*
*Tim17b*	*F 5′ ACCTTGCCCTTACAGAATAGTTG 3′*
	*R 5′ ATGCTTCCCACCAATCTTCTAT 3′*
*Dmel\CG6733*	*F 5′ GGTGCATCCAGATGTTGACTATAC 3′*
	*R 5′ TTCGGGTTGACTTCCTTCC 3′*
*SLIRP1*	*F 5′ AATCTACCGTGGACAGTGGG 3′*
	*R 5′ CAGTATGTGCTTCTGCTCGTTC 3′*
*GIP*	*F 5′ TGATTTCGCCCGACAGGT 3′*
	*R 5′ CCAGCGGCTTTGGAGTATG 3′*
*sPLA2*	*F 5′ GGACGAGGACATCTACAACCA 3′*
	*R 5′ CACTTGTCCGTCTCCCGTT 3′*
*Pebp1*	*F 5′ CGCTACGTCTTCCTGGTGTT 3′*
	*R 5′ GGACCGCCGAAGCTGTA 3′*
*Cyp6t3*	*F 5′ CTGCTCATCTGGCTGCTGTT 3′*
	*R 5′ CTTCGCCTGACCATTTCGT 3′*
*spartin*	*F 5′ ACATTGTAAGTGCGGCTGATT 3′*
	*R 5′ GCTGGCGTCATCTTGGAA 3′*
*Cyp6a18*	*F 5′ ACAGTCGGTCATTATTCCATCA 3′*
	*R 5′ GCCTGCATCTGTCCAAATCT 3′*

### 2.8 Western blotting

Female *D melanogaster* (36 days old, 20 flies each) of the control and RG groups was anaesthetized and collected. Pebp1, spartin, Ent2, CG9062, Tim17b and TSG101 differentially expressed proteins were selected for verification using Western blotting. The method referred to the lab's previous approach.^1^ GAPDH (Proteintech) was used as a loading control.

### 2.9 Statistical analyses

The comparison was made using Student's *t*‐test. A *P* value of < .05 was considered statistically significant.

## 3 RESULTS

### 3.1 Lifespan analysis of female *Drosophila melanogaster*

**Figure 1:**
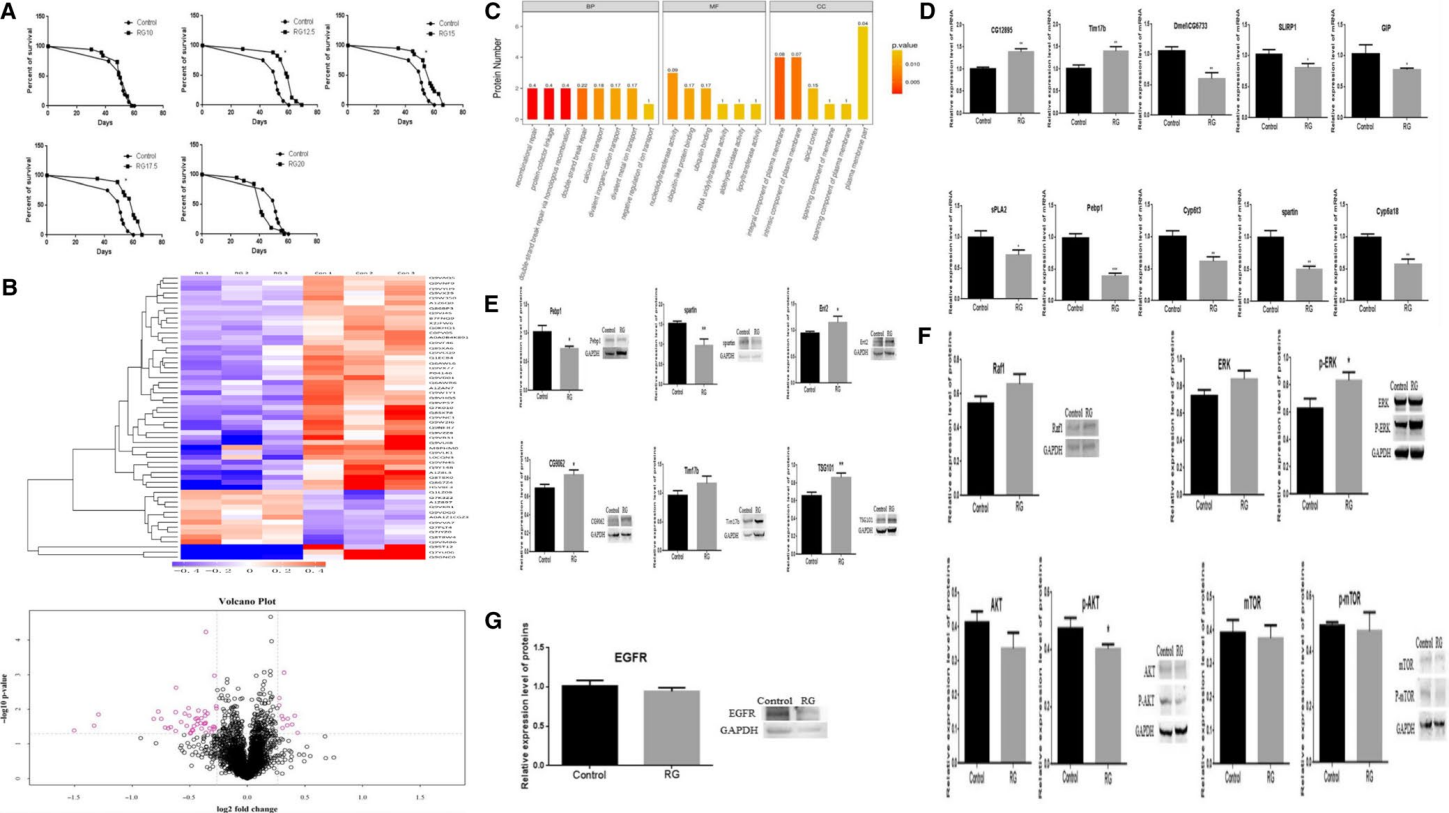
Effect of RG on the lifespan of *Drosophila melanogaster*. A, Effects on the lifespan of RG‐treated *D melanogaster*. B, The identified differentially expressed proteins by using clustering heatmaps and volcano plot. C, GO term annotation analysis of the differential proteins. D, Validation of differential proteins by qRT‐PCR. E, Expression levels of Pebp1, spartin, Ent2, CG9062, Tim17b and TSG101 were confirmed using Western blotting. F, Expression levels of Raf1, ERK, p‐ERK, AKT, p‐AKT, mTOR and p‐mTor were confirmed using Western blotting. G, Expression levels of EGFR were confirmed using Western blotting. Significance analysis was indicated by student's *t*‐test (**P < .01, *P < .05). RG, red ginseng; Con, control; Pebp1, phosphatidyl ethanolamine‐binding protein 1; AKT, protein kinase B; ERK, extracellular regulated protein kinases; mTOR, mechanistic target of rapamycin; EGFR, epidermal growth factor receptor

Treatment with 10 mg/mL RG did not significantly extend the lifespan of female *D melanogaster*, while treatment with 12.5, 15 and 17.5 mg/mL RG significantly extended the lifespan of the flies (*P* = .0356, .0426 and .0015). However, 20 mg/mL RG reduced the lifespan of female *D melanogaster* (Figure 1A).

### 3.2 Identification of differentially expressed proteins

Using a threshold of >1.2 or <0.83 (*P* ≤ .05), 11 proteins were up‐regulated and 46 proteins were down‐regulated. Clustering heatmaps and volcano plot were used to identify significant changes in protein expression (Figure 1B).

### 3.3 Bioinformatics analysis of differentially expressed proteins

The differentially expressed proteins were subjected to gene ontology (GO)‐based enrichment analysis using the Blast2GO program and Fisher's exact Test (*P* ≤ .05; Figure 1C).

### 3.4 Validation of differentially expressed proteins by qRT‐PCR

To explore the expression changes of the differentially expressed proteins during transcription or translation, the mRNA levels of these proteins identified by iTRAQ was detected by qRT‐PCR. Compared with the protein changes from iTRAQ analysis, the variations of CG12895 (*P* = .0011), Tim17b (*P* = .0061), Dmel\CG6733 (*P* = .0028), SLIRP1 (*P* = .0207), GIP (*P* = .0409), sPLA2 (*P* = .0198), Pebp1 (*P* = .0002), Cyp6t3 (*P* = .0038), spartin (*P* = .0018) and Cyp6a18 (*P* = .0015) at mRNA level were consistent with the protein analysis (Figure 1D).

### 3.5 Validation of proteins by Western blotting

Expression levels of Pebp1, spartin, Ent2, CG9062, Tim17b and TSG101 were explored through Western blotting. The altered expressions of Pebp1 (*P* = .0131), spartin (*P* = .0047), Ent2 (*P* = .0449), CG9062 (*P* = .0189) and TSG101 (*P* = .0054) in the RG group compared to the control group were consistent with the results of iTRAQ (Figure 1E).

### 3.6 Effect of Pebp1 on the ERK pathway and AKT/mTOR pathway

Based on the experimental results of iTRAQ, qRT‐PCR and Western blotting, Pebp1 was identified as an important protein among the identified differentially expressed proteins. Pebp1 is an inhibitor of Raf1, a kinase of the Raf/MEK (MAP/ERK kinase)/ERK signalling pathway, and mainly plays a role in cell proliferation and differentiation.^6^, ^7^ Moreover, Pebp1 reportedly regulates many other signalling pathways, such as AKT/mammalian target of rapamycin (mTOR).^8^ We assessed the protein levels of Raf1, ERK, phosphorylated (p)‐ERK, AKT, p‐AKT, mTOR and p‐mTOR by Western blotting. Compared with the control group, RG treatment down‐regulated the levels of AKT and p‐AKT (*P* = .0412), and up‐regulated the levels of ERK, p‐ERK (*P* = .0204), and Raf1; in contrast, the expression levels of mTOR and p‐mTOR remained unchanged (Figure 1F).

## 4 DISCUSSION

RG prolongs the lifespan of *D melanogaster*, but its mechanism is yet to be elucidated. In this study, protein changes in 36‐day‐old (old‐age) female *D melanogaster* were analysed by iTRAQ. The iTRAQ examination revealed 11 proteins were up‐regulated and 46 were down‐regulated. qRT‐PCR and Western blotting provided verification.

**Figure 2:**
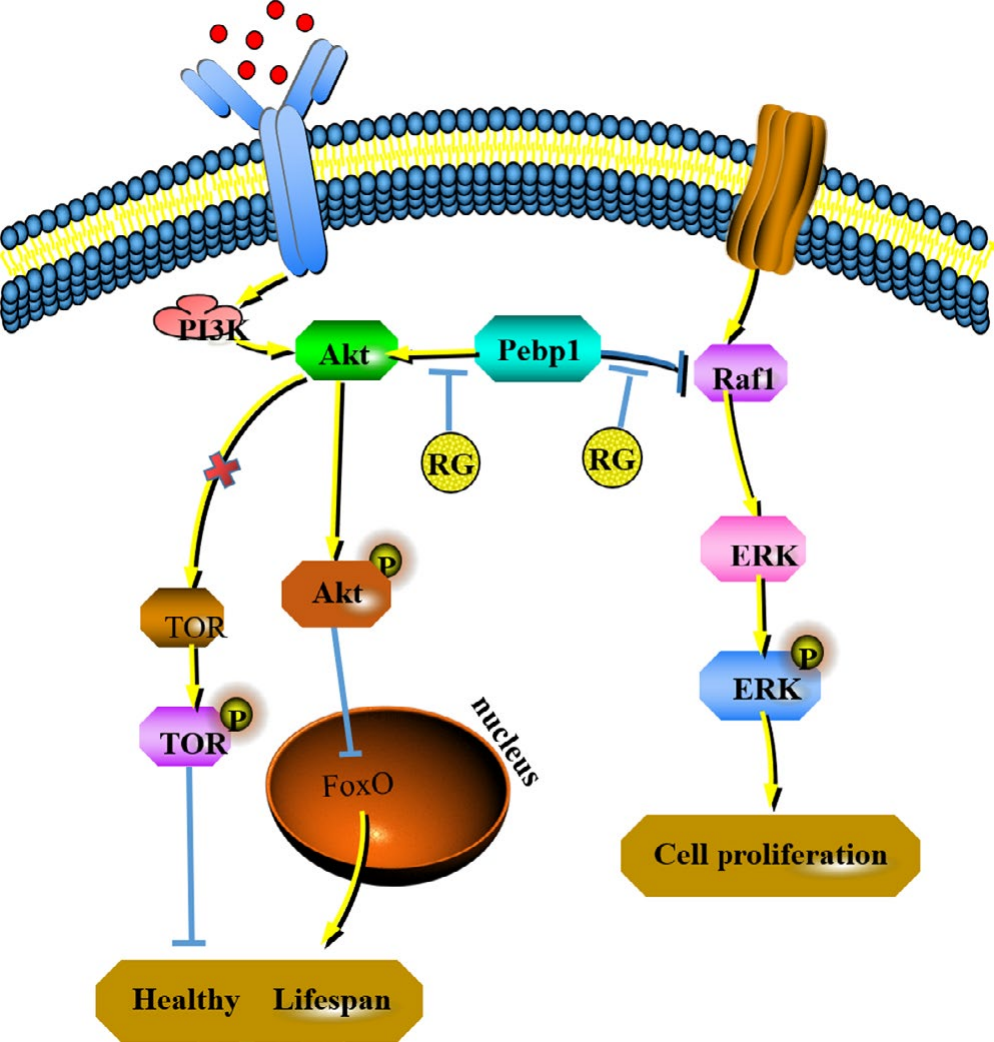
The possible mechanism of anti‐ageing effects induced by RG in *Drosophila melanogaster*. RG, red ginseng; Con, control; Pebp1, phosphatidyl ethanolamine‐binding protein 1; AKT, protein kinase B; ERK, extracellular regulated protein kinases; mTOR, mechanistic target of rapamycin; EGFR, epidermal growth factor receptor

Pebp1 inhibits the Raf‐MAPK/ERK pathway by directly blocking Raf1 activation.^8^ Western blotting results showed that the level of Pebp1 protein in female *D melanogaster* was reduced after RG treatment and its interaction with Raf1 decreased. Compared with the control group, the ERK pathway was up‐regulated in the RG group, which promoted the proliferation of senile cells. Elsewhere, the overexpression of Pebp1 has been reported to stimulate the AKT/mTOR pathway.^8^ Our results showed that RG treatment down‐regulated the levels of AKT and p‐AKT, while not changing the levels of mTOR and p‐mTOR. These findings indicate that RG treatment may reduce the level of Pebp1, and thus, inhibit the activity of AKT signalling pathways. Inactivation of AKT directly activates the FoxO family and prolongs the lifespan.^9^ The role of the AKT/FoxO signalling in longevity appears to be conserved across species.^10^ These collective findings indicate that RG prolonged the lifespan of female *D melanogaster* by reducing the expression of Pebp1, up‐regulating ERK and inhibiting the AKT pathway (Figure 2).

The iTRAQ and qRT‐PCR analyses revealed the highly significant variation of spartin. Spartin is present in organisms ranging from nematodes and flies to humans,^11^ and it participate in endocytosis by interacting with epidermal growth factor receptor pathway substrate 15 (Eps15).^12^ Knockout or overexpression of Spartin leads to a decrease in the degradation rate of epidermal growth factor receptor (EGFR), which affects the internalization of EGFR. EGFR degradation impairs endocytosis and synaptic growth, which may underlie the pathogenesis of Troyer syndrome.^13^, ^14^ Presently, Western blotting revealed the significantly lower level of spartin in the RG group compared to the level in the control group, with EGFR level remaining the same in the RG group (Figure 1G). We suggest that EGFR is normally degraded or recycled without affecting downstream signalling pathways. Therefore, integrated iTRAQ, qRT‐PCR and Western blotting analyses were used to comprehensively analyse the protein changes in 36‐day‐old (old‐age) female *D melanogaster*. RG significantly prolonged the lifespan of the flies by reducing the expression of Pebp1, up‐regulating ERK and inhibiting the AKT pathway. Therefore, RG may be a potential drug for anti‐ageing treatment.

## CONFLICT OF INTEREST

The authors declare that they have no conflicts of interest.

## AUTHOR CONTRIBUTIONS

WH and JP conceived and designed the experiments. WH, YPW and JZ contributed to the acquisition of data. HSZ analysed and interpreted the data. WH, JP and RJC contributed to drafting the article. All authors have revised the manuscript critically for important intellectual content and approved the final version to be published.

## Data Availability

Research data are not shared.
